# Case report: IgG4-related intracranial lesions mimicking multiple sclerosis in a 14-year-old girl

**DOI:** 10.3389/fneur.2022.1007153

**Published:** 2022-09-28

**Authors:** Pingying Qing, Chenyang Lu, Bing Yan, Chang Liu, David A. Fox, Yi Zhao, Yi Liu, Chunyu Tan

**Affiliations:** ^1^Department of Rheumatology and Immunology, West China Hospital, Sichuan University, Chengdu, China; ^2^Laboratory of Rheumatology and Immunology, West China Hospital, Sichuan University, Chengdu, China; ^3^Department of Radiology, West China Hospital, Sichuan University, Chengdu, China; ^4^Division of Rheumatology, Clinical Autoimmunity Center of Excellence, University of Michigan Medical School, Ann Arbor, MI, United States

**Keywords:** multiple sclerosis, IgG4-related disease, magnetic resonance imaging, brain parenchyma, lymphadenopathy

## Abstract

**Objectives:**

IgG4-related disease (IgG4-RD) is distinguished by the infiltration of IgG4-positive plasma cells in a variety of tissues and organs. Even so, central nervous system lesions associated with IgG4-RD are scarce. We present a case of IgG4-related brain parenchymal lesions that mimics multiple sclerosis in a young girl.

**Methods:**

The patient was followed by our neurology and rheumatology teams. Clinical information was recorded, and the brain was screened using magnetic resonance imaging (MRI). During follow-up, we examined serum IgE, IgG and IgG4 and lymph node biopsy.

**Results:**

Here, we presented details of a 14-year-old Chinese girl suffering from diplopia, left eyelid ptosis, right facial numbness, and right lower limb weakness admitted to our institute. Brain MRI revealed multiple sclerosis-like lesions in the brain parenchyma and spinal cord. During the follow-up, she developed lymphadenopathy. Elevation of serum, IgG, IgG4 and IgE and lymph node biopsy favors a diagnosis of IgG4-RD. The patient had a good response to glucocorticoids and mycophenolate mofetil. The literature review summarized eight previously reported IgG4-RD involving brain parenchyma.

**Discussion:**

Our case expands the known age spectrum of IgG4-RD. The intracranial IgG4-RD is rare and could mimic multiple sclerosis. Careful examination and dynamic review of disease history are crucial in the differential diagnosis.

## Introduction

IgG4-related disease (IgG4-RD) is a syndrome that forms an inflammatory pseudotumor with densely infiltrated lymphocytes, including IgG4^+^ plasmacytes along with storiform fibrosis and obliterative phlebitis (1, 2). The disease typically affects middle-aged and older people (55–59 years), preferentially prevalent more in men (1). It can involve virtually every organ in the body, including the lacrimal glands, the salivary glands, the pancreas, the biliary tree, the kidneys, and the retroperitoneum (2). However, intracranial IgG4-RD is rare and can present as a single pseudotumor or diffused lesion in the substance of the dura matter, the pia matter, the pituitary gland and stalk, and less likely brain parenchyma (3). Multiple sclerosis (MS) is a chronic inflammatory demyelinating disease of the central nervous system (CNS) characterized by heterogeneity in clinical symptoms. MS plaques on magnetic resonance imaging (MRI) appear as multiple, well-demarcated, homogenous, small ovoid lesions lacking mass effect and are often oriented perpendicular to the long axis of the lateral ventricles (4). Differential diagnoses of IgG4-RD and MS can be challenging for both clinicians and radiologists as both diseases have various manifestations and lack specific markers. Here, we present a young girl with IgG4-related intracranial lesions that mimic multiple sclerosis (MS).

## Case description

**Figure 1:**
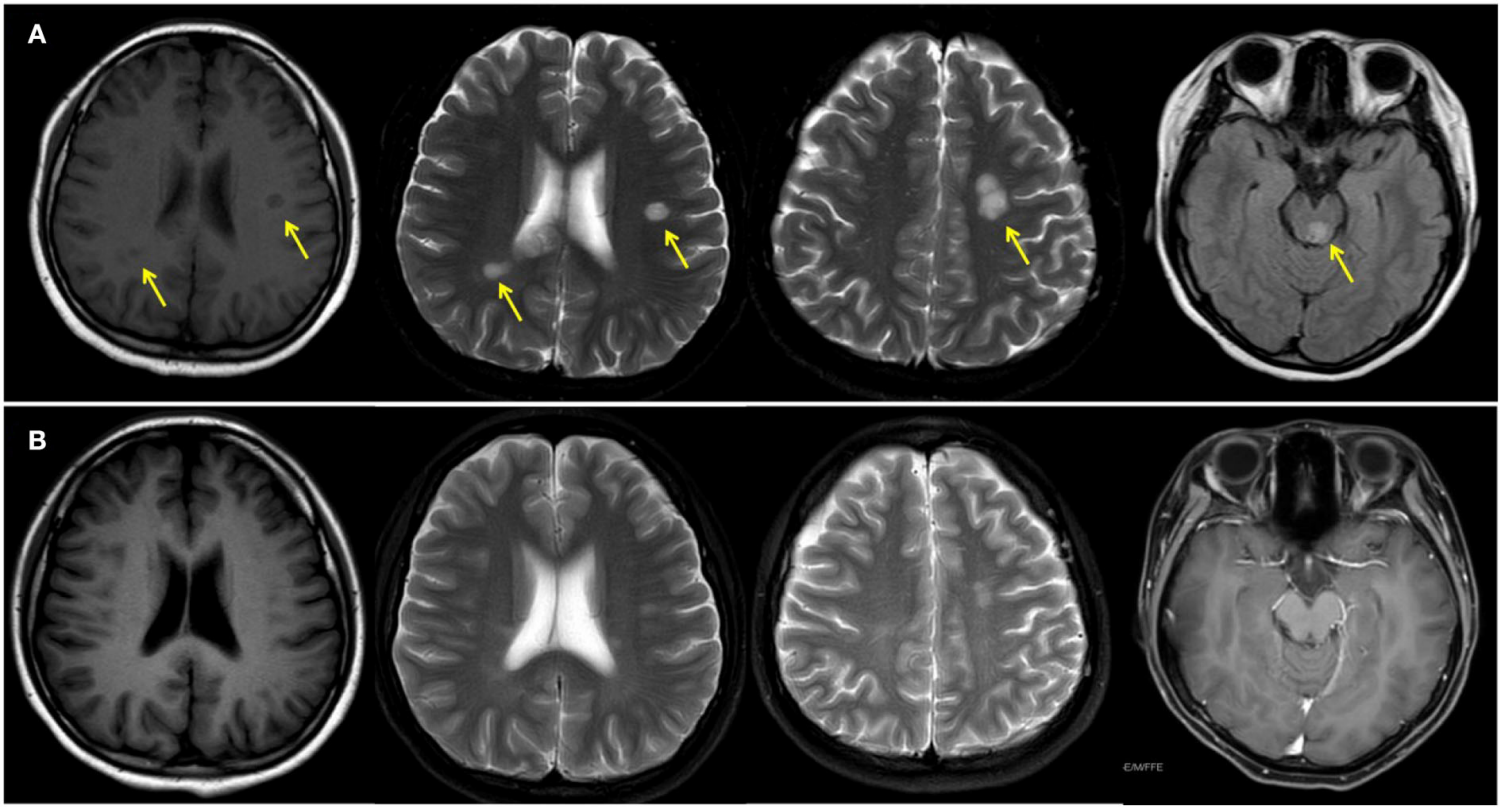
Magnetic resonance imaging of the brain before **(A)** and after **(B)** treatment. Yellow arrows indicate white matter lesions.

**Figure 2:**
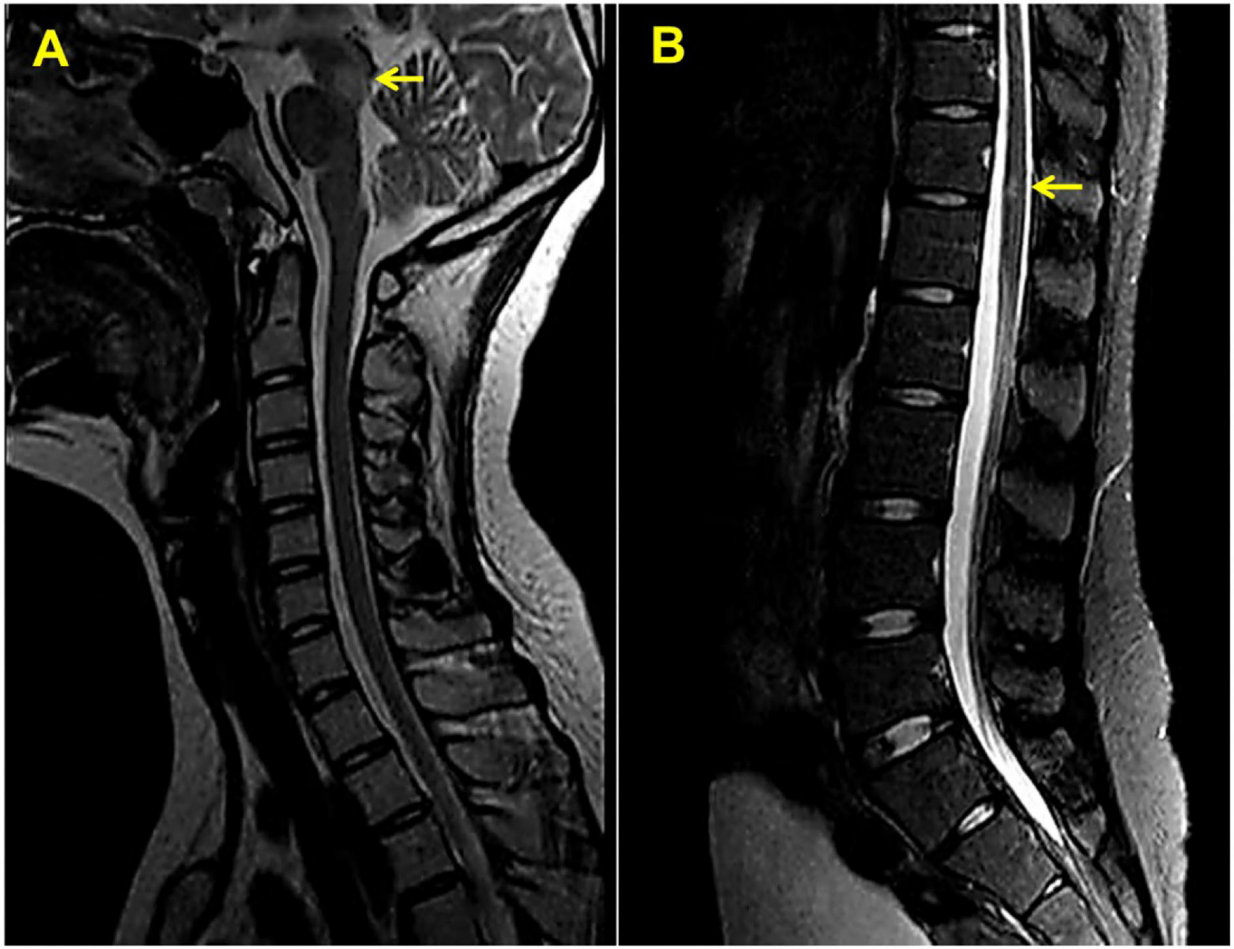
Sagittal T2-weighted magnetic resonance imaging of the upper **(A)** and lower **(B)** spinal cord. Yellow arrows indicate white matter lesions.

**Figure 3:**
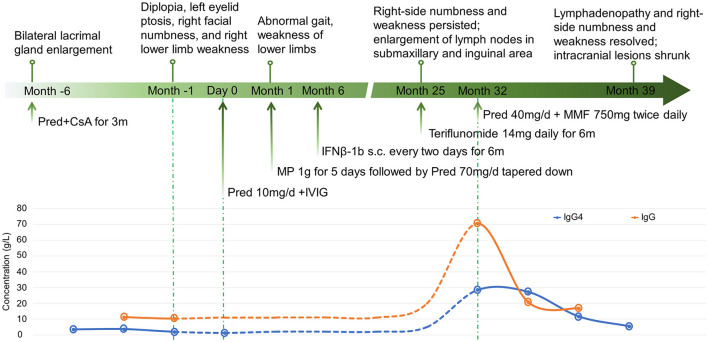
The disease course, treatments, and the changes of serum IgG and IgG4 of the patient.

**Figure 4:**
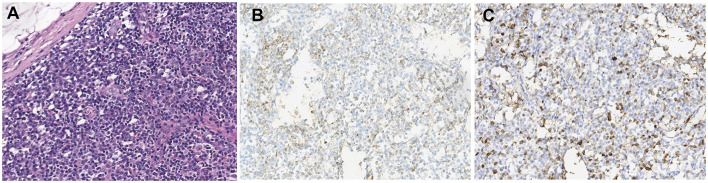
Histological examination of the biopsy of inguinal lymph node **(A)** H&E staining; **(B)** immunohistochemistry for CD138; **(C)** immunohistochemistry for IgG4; 200×).

A 14-year-old girl presented with diplopia, left eyelid ptosis, right facial numbness, and right lower limb weakness since 1 month prior, which had progressively worsened. Physical examination revealed an impaired vision of both eyes, diplopia, left eyelid ptosis, right facial paresthesia, and hyperreflexia of both sides. There were no meningeal signs and no cervical, axillary, or inguinal lymphadenopathy. Routine blood tests, including blood cell counts, albumin level, transaminases, and C-reactive protein, were not significant. Brain MRI revealed multiple coin-like white matter lesions and one mesencephalon lesion (Figure 1A). Sagittal T2-weighted imaging of the brainstem and the spinal cord showed an enhanced lesion in the cerebral peduncle (Figure 2A) and in the spinal cord at the level of thoracic 12 (Figure 2B). Cerebral spinal fluid analysis showed leukocytes within the normal range, glucose was 6.25 mmol/L, albumin was 0.128 g/L, total Ig was 0.0563 g/L, IgG synthesis rate was 0 mg/day, and there were no detectable oligoclonal bands. The visual evoked potential (VEP) test was normal. She had a history of bilateral lacrimal gland enlargement about 6 months before, and a surgery was performed to correct this. Histological examination indicated lymphoproliferative changes, and the number of IgG4^+^ cells was over 220/HPF. Serum IgG4 was elevated (3.45 g/L, normal range 0.049–1.35 g/L). The patient was diagnosed as “IgG4-RD” and received oral glucocorticoid combined with cyclosporin but stopped 3 months later. The patient's family history was not remarkable. Based on the symptoms and MRI findings, the patient was suspected of MS and was given prednisone and intravenous immunogloblin (IVIG). Her diplopia and ptosis improved significantly and was discharged. One month later, she was administered 1 g of methylprednisolone for 5 days, followed by 70 mg of prednisone daily since her abnormal gait and lower limb weakness persisted. The prednisone was tapered to stop while recombinant interferon (IFN) β-1b was given every 2 days subcutaneously for 6 months. She did not receive any treatment for over 1 year until the development of enlargement of lymph nodes in the submaxillary and inguinal areas. Fourteen milligrams of Teriflunomide was given daily; however, her symptoms persisted. Six months later, the patient came to our department with lymph node enlargement and right-side numbness and weakness, but she denied any fever, unintentional weight loss, fatigue, or change in appetite. Laboratory tests revealed normal counts of leukocytes and platelets, the hemoglobin level, the albumin level, the serum creatinine level, and C-reactive protein. Antinuclear antibodies, extractable nuclear antigen antibodies, anti-neutrophil cytoplasmic antibodies, and anti-phospholipid antibodies were all negative. Serum IgG was 70.7 g/L (normal range 8.0–15.5 g/L), IgG4 was 28.5 g/L, and IgE was 4,810 mg/L (normal range 5–150 mg/L), while IgA and IgM were within the normal range (Figure 3). Chest computerized tomography and abdominal ultrasonography were not significant. A biopsy of the left inguinal lymph node was performed. The histopathological evaluation of the biopsy specimen of the inguinal lymph node revealed mixed inflammation containing predominantly plasma cells (Figures 4A–C). No granuloma, prominent necrosis, “onion skin pattern” mantle zones, or “lollipop lesions” were found. Immunostaining showed an increased number of IgG4^+^ plasma cells (>200/HPF) and an elevated ratio of IgG4^+^ cells to CD138^+^ plasma cells (~80%) (Figures 4B,C). No mycobacteria, fungi, or parasites were noticed, and Epstein-Barr virus-encoded RNA 1/2 was negative. These findings were suggestive of an IgG4-RD involving the lacrimal glands, the brain parenchyma spinal cord, and the lymph nodes according to both the ACR/EULAR classification criteria (5) and the revised comprehensive criteria (6). She had a good response to oral prednisone 40 mg daily combined with mycophenolate mofetil 750 mg two times daily. In a consecutive 7-month follow-up, her lymphadenopathy and right-side numbness and weakness resolved. A repeated brain MRI revealed shrinkage of the intracranial lesions, and the lab tests showed a rapid drop in serum levels of IgG and IgG4 (Figures 1B, 3).

## Discussion

Our case illustrates an intracranial IgG4-RD mimicking MS in a 14-year-old girl, which has been rarely reported in the literature. In this case, it is important to make differential diagnoses among some relevant diseases including CNS tumor, infectious encephalitis, and immune-mediated encephalitis, such as MS.

Some mimickers, especially idiopathic multicentric Castleman's disease (CD), are sometimes difficult to be differentiated from IgG4-RD (7) and should be excluded before a diagnosis was made. The patient did not have a fever, large spleen or liver, serositis, angioma, or interstitial pneumonitis. Lab tests did not reveal anemia, thrombocytopenia, hypoalbuminemia, elevated CRP, or renal dysfunction. Elevated IgG and IgE but not IgA and IgM were found in the serum. The lymph node biopsy did not reveal “onion skin pattern” mantle zones, “lollipop lesions,” or sheet-like mature plasma cell proliferation, which are characteristic findings of CD (8), and the enlarged lymph nodes resolved quickly with glucocorticoids. Therefore, the CD can be ruled out. Moreover, the negative autoantibody spectrum did not support other autoimmune diseases such as primary Sjogren's syndrome and systemic lupus erythematosus, whereas benign clinical features and histological findings ruled out lymphoma. Based on localized swelling of the lacrimal glands and the lymph nodes, elevated IgG4 level, and characteristic histological findings, the patient received a diagnosis of IgG4-RD.

IgG4-RD can affect almost every organ system, where 31% of patients were affected by hepato-pancreato -biliary diseases, 24% by retroperitoneal fibrosis and aortitis, 24% by head and neck-limited disease, and 22% by classic Mikulicz syndrome with systemic involvement (9). Nearly 40% of patients present with a clinically-evident disease in a single organ system, and it may evolve overtime, with new systems being involved in a metachronous manner (10). This patient showed different organ involvement at different stages, i.e., swelling of bilateral lacrimal glands at the beginning followed by intracranial and spinal cord lesions and recent lymph node enlargement, suggesting the complexity of this disease.

IgG4-RD can also involve the nervous system, including the central nervous system (CNS) and the peripheral nervous system. Neurological IgG4-RD has two main mechanisms: infiltration in the substance of the nervous system and compression of neurological structures by the mass effect of nearby diseased tissues (3). It most commonly manifests in the substance of the dura matter, followed by the pituitary gland and stalk, the peripheral nerves, the brain parenchyma, and occasionally the pia matter (3). So far, only a handful of cases of IgG4-RD involving brain parenchyma are reported (Supplementary Table 1). In these cases, most patients were middle-aged men (17–62 years old). Their neurological symptoms varied a lot, depending on the location and area of the intracranial lesions. The IgG4-related lesions were most likely found in the periventricular area or close to the meninges with MRI (Supplementary Table 1). To the best of our knowledge, this case, which starts to get symptoms at 13-year-old, is the youngest patient with histologically confirmed IgG4-RD.

Neural symptoms and white matter plaques on MRI of this patient (Figure 1) usually prompt people to consider MS. MS attacks myelinated axons in the CNS, causing progressive neurological deterioration (11). This disorder usually presents in adults between the age of 20 and 45 years with female predominance (11). Its clinical symptoms are non-specific and usually include visual changes, numbness, weakness, and paralysis, depending on the amount and area of nerve damage. MS is usually diagnosed by demonstrating clinical and/or radiographic evidence of dissemination of disease in time and space (12). This disease is characterized by elliptical or ovoid lesions found in the white matter of the periventricular and juxtacortical regions, the cerebellar peduncles, the superficial pons, and the floor of the fourth ventricles (13). Spinal cord lesions are also commonly seen. T2-weighted imaging of the spinal cord demonstrates small and circumscribed high-signal lesions that are aligned with the long axis of the cord. The lesions are usually less than two vertebral segments in length and involve less than half the axial cord area (13). In this patient, MRI revealed multiple ovoid lesions in the white matter and mesencephalon, and an enhanced lesion in the spinal cord at the level of thoracic 12, which are similar to MS lesions. Because cerebral IgG4-RD and MS have distinct outcomes, distinguishing between these two entities is critical. However, clinical judgment is often required to make the classification given an absence of specific biomarkers and clear diagnostic criteria.

In addition to MS, the differential diagnosis of pseudo-tumoral brain lesions includes infection neoplastic, including primary CNS lymphomas and metastatic cancers; congenital, metabolic, or vascular diseases; and non-MS idiopathic inflammatory demyelinating diseases, which include neuromyelitis optica (NMO) spectrum disorders (NMOSD), opticospinal MS, and acute disseminated encephalomyelitis. NMOSD is characterized by simultaneous or consecutive attacks of acute optic neuritis and transverse myelitis (14). IgG autoantibodies against aquaporin 4 (AQP4) or myelin oligodendrocyte glycoprotein are widely present in these patients. In AQP4 positive NMOSD, whiter matter lesions are typically large, confluent, unilateral or bilateral subcortical and deep. Sometimes both brain and brainstem lesions can occur bilaterally, and brain lesions tend to be longitudinally extensive, involving the corticospinal tract and corpus callosum. Myelitis can manifest as a longitudinally intramedullary spinal lesion that extends over three vertebral segments. More than 50 cells/ul of white cell counts are frequently found in CSF of NMO. Prolonged P100 latencies in VEP were also present in 42–72% of NMOSD (14). The patient did not have an anti-AQP4 antibody test; however, the normal white cell counts in CSF and normal VEP with small and circumscribed lesions in the brain parenchyma and spinal cord on MRI can rule out the diagnosis of NMO. This patient had IgG4-RD involving the brain parenchyma, the lacrimal glands, and the lymph nodes, with distinct organ involvement present at different stages. Careful examination and dynamic review of medical history favor accurate diagnosis.

Similar to MS, the exact pathogenesis of IgG4-RD remains elusive but aberrant innate and adaptive immunity are considered to be involved (15, 16). B cells have been demonstrated to play a central role in IgG4-RD, while CD4+ cytotoxic T lymphocytes and T follicular helper cells contribute to the IgG4 isotype switching (16, 17). IgG4-RD is highly treatable with corticosteroids, but almost 40% either fail to achieve complete remission or relapse within 1 year even with a low dose of glucocorticoid (16). Some disease-modifying anti-rheumatic drugs (DMARDs), such as azathioprine and mycophenolate mofetil, were able to increase the rate of inducing remission and reduce disease flares (1, 10, 18). Other conventional steroid-sparing medications are also effective in this disease, but data are limited to retrospective analyses or case reports. B-cell depletion therapy including rituximab can usually leads to disease remission in most cases, allowing early tapering of glucocorticoid therapy (19). Because IgG4-RD and MS have some common immunological abnormalities, several immunosuppressive treatments are shared between these two entities. In MS, immune cell activation and the blood–brain barrier breakdown cause demyelination and axon injury. IFN-β can improve the disease course of MS by reducing antigen presentation, reducing T-cell proliferation, decreasing the expression of cytokines, and reducing matrix metalloproteinase (20). However, a sustained elevation of type I IFN may accompany clinical manifestations and disease activity in systemic autoimmune disease, including systemic lupus erythematosus, primary Sjogren's syndrome, and systemic sclerosis (21). On the contrary, IFN-β was proved to be highly effective in a lupus mouse model (22). There is a strong IFN-γ signal in IgG4-RD immune cells (23); however, the role of type I IFN in the pathophysiology and outcome of the disease remains unknown. In this case, the disease relapsed during the usage of IFN-β-1b and discontinuance of prednisone. It is difficult to identify the causes of the disease flare.

This case report in conjunction with those in the literature indicates that IgG4-RD represents a great mimicker of many neoplastic, inflammatory, and infectious conditions. Histopathology remains critical to diagnosis because reliable biomarkers are lacking. IgG4-RD responds promptly to glucocorticoids and DMARDs, but prolonged courses are often needed because the disease relapses in most patients. This case illustrates the importance of dynamic monitoring and pathological examination to distinguish cerebral IgG4-RD from other CNS disorders and underscores the need for clear clinical guidelines to establish a diagnosis of IgG4-RD.

## Data availability statement

The original contributions presented in the study are included in the article/Supplementary material, further inquiries can be directed to the corresponding authors.

## Ethics statement

This study was approved by the Ethics Committee of West China Hospital of Sichuan University. Written informed consent was obtained from the patient and her parent for the publication of any potentially identifiable images or data included in this article.

## Author contributions

PQ and BY collected the clinical information. CLiu interpreted the brain MRI findings. CLu made literature review and drafted the article. DF, YZ, YL, and CT interpreted the data and revised the article. All authors contributed to the article and approved the submitted version.

## Funding

This study was supported by the National Natural Science Foundation of China (Nos. 81501412 and 82104484), the Natural Science Foundation of Sichuan Province (2022NSFSC1542), the Post-Doctor Research Project, West China Hospital, Sichuan University (2021HXBH014), West China Hospital, Sichuan University 1.3.5 Project for Disciplines of Excellence, and West China Hospital, Sichuan University (No. ZYGD18015).

## Conflict of interest

The authors declare that the research was conducted in the absence of any commercial or financial relationships that could be construed as a potential conflict of interest.

## Publisher's note

All claims expressed in this article are solely those of the authors and do not necessarily represent those of their affiliated organizations, or those of the publisher, the editors and the reviewers. Any product that may be evaluated in this article, or claim that may be made by its manufacturer, is not guaranteed or endorsed by the publisher.
