# Supplementary material for: Case report: IgG4-related intracranial lesions mimicking multiple sclerosis in a 14-year-old girl
Source: Front Neurol. 2022 Sep 28;13:1007153. doi: 10.3389/fneur.2022.1007153 (PMC9554464; doi:10.3389/fneur.2022.1007153)
Supplement: Supplementary file 1 [file Table_1.DOCX]

**Supplementary Table 1 Summary of IgG4-RD patients involving brain parenchyma**

| **Author and year** | **Sex and age (y)** | **Neurological Presentations** | **Brain MRI findings** | **CSF examination** | **Other symptoms and diagnosis** | **Serum IgG4 (mg/dl)** | **Histology** | **Treatment and outcome** |
| --- | --- | --- | --- | --- | --- | --- | --- | --- |
| Kim EH, 2011(1) | Male, 43 | Headache and progressive motor weakness in the right upper extremity | Enhanced nodular mass near the corpus callosum and cingulate gyrus | NA | Diabetes mellitus, hypertension |  | Mass: thickened fibrous tissue with marked inflammatory cell infiltration. IgG4^+^/IgG^+^ =94% | Surgery followed by prednisolone 30mg/d, improved |
| Regev K, 2014(2) | Male, 50 | Left spastic hemiparesis, left hemimyoclonus, and cognitive decline. | 3 white matter lesion and 1 right frontal cortical lesions were hyperintense on T2 and fluid-attenuated inversion recovery sequence | Elevated total protein | Enlarged parotid gland, and elevated liver enzymes, type 1 diabetes, exocrine type 1 pancreatic insufficiency | 411 | Meninges: plasma cells (CD138^+^) > 10% of the immune cells, and most were IgG4+ | Higd dose methylprednisolone for 9 dyas and then oral prednisone 60mg/d, recovered |
| Li L, 2015(3) | Female, 58 | Left lower limb weakness | An enhancing lesion at the falx and eroded into the right frontal lobe | NA | NA | 76.8  (normal) | Falx cerebrii: fibroinflammatory lesion with fibrosis mixed with lymphocytes and plasma cells. IgG4^+^/IgG^+^ =40% | Surgery and dexamethasone, improved. |
| Joshi D, 2015(4) | Male, 56 | Confusion, decreased consciousness | Progressive diffuse periventricular and subcortical white matter signal change | normal | Enlarged submandibular salivary gland. Intrahepatic biliary dilation, pancreatic enlargement, gastric and small bowel wall thickening, and mesenteric lymphadenopathy | 230 | Salivary gland: >10 IgG4^+^ plasma cells per hpf | High dose steroid, died 11 months later |
| Tanji H, 2016(5) | Female, 58 | Right hand clumsiness, speaking difficulty, sensory aphasia, right hemiparesis | Extensive signal abnormalities in the left hemisphere extends from the internal capsule to cerebral peduncle | Increased protein concentration with no pleocytosis | Pancytopenia, liver dysfunction, sclerosing cholangitis | 261 | Brain: lymphocyte and plasma cell infiltration and reactive gliosis | Betamethasone 8mg/d, followed by tapered prednisone from 60mg/d to 15mg/d. Improved. |
| Zhang Z, 2018(6) | Male, 29 | Binocular temporal visual field defect, memory loss, headache | Progressive multiple abnormal signals in the brain parenchyma, surrounding by edema | NA | Liver dysfunction | 304 | NA | Methylprednisolone 80mg/d🡪40mg/d. Improved |
| Vakrakou AG, 2020(7) | Female, 17 | Numbness and paresthesia in upper limbs, trunk, and lower limbs | Hyperintensity lesions in the deep white matter, enlargement of hypophysis | normal | Longitudinal intramedullary damage of the cervical spinal cord | 146 | NA | Methylprednisolone 1g for 5 days followed by glucocorticoid and azathioprine (2mg/kg). Improved. |
| Temmoku J, 2020(8) | Male, 62 | Drowsiness, urinary retention, and fecal incontinence | Scattered hyerintense signals in the left hypothalamus, bilateral insula, and putamen | Increased protein concentration with no pleocytosis | Lymphadenopathy, mild normocytic anemia and liver dysfunction | 1490 | Lymph node: massive infiltration of lymphocytes and plasma cells. IgG4^+^/IgG^+^ plasma cells nearly 100% | Methylprednisolone 1g for 3 days followed by oral prednisolone 1mg/kg, improved |

**References**

1. Kim EH, Kim SH, Cho JM, Ahn JY, Chang JH. Immunoglobulin G4-related hypertrophic pachymeningitis involving cerebral parenchyma. J Neurosurg. 2011;115:1242-7.

2. Regev K, Nussbaum T, Cagnano E, Giladi N, Karni A. Central nervous system manifestation of IgG4-related disease. JAMA Neurol. 2014;71:767-70.

3. Li LF, Tse PY, Tsang FC, Lo RC, Lui WM, Leung GK. IgG4-Related Hypertrophic Pachymeningitis at the Falx Cerebrii with Brain Parenchymal Invasion: A Case Report. World Neurosurg. 2015;84:591 e7-10.

4. Joshi D, Jager R, Hurel S, Pereira SP, Johnson GJ, Chapman M, et al. Cerebral involvement in IgG4-related disease. Clin Med (Lond). 2015;15:130-4.

5. Tanji H, Okada H, Igari R, Yamaguchi Y, Sato H, Takahashi Y, et al. Inflammatory Pseudotumor of the Brain Parenchyma with IgG4 Hypergammaglobulinemia. Intern Med. 2016;55:1911-6.

6. Zhang Z, Fu W, Wang M, Niu L, Liu B, Jiao Y, et al. IgG4-related inflammatory pseudotumor of the brain parenchyma: a case report and literature review. Acta Neurol Belg. 2018;118:617-27.

7. Vakrakou AG, Evangelopoulos ME, Boutzios G, Tzanetakos D, Tzartos J, Velonakis G, et al. Recurrent myelitis and asymptomatic hypophysitis in IgG4-related disease: case-based review. Rheumatol Int. 2020;40:337-43.

8. Temmoku J, Sato S, Matsumoto H, Fujita Y, Suzuki E, Yashiro-Furuya M, et al. IgG4-Related Disease Complicated by Brain Parenchymal Lesions Successfully Treated with Corticosteroid Therapy: A Case Report. Tohoku J Exp Med. 2020;251:161-8.
